# Biodistribution and Biosafety of a Poly(Phosphorhydrazone) Dendrimer, an Anti-Inflammatory Drug-Candidate

**DOI:** 10.3390/biom9090475

**Published:** 2019-09-11

**Authors:** Séverine Fruchon, Elisabeth Bellard, Nicolas Beton, Cécile Goursat, Abdelouahd Oukhrib, Anne-Marie Caminade, Muriel Blanzat, Cédric-Olivier Turrin, Muriel Golzio, Rémy Poupot

**Affiliations:** 1 INSERM, U1043, CNRS, U5282, Université de Toulouse, UPS, Centre de Physiopathologie de Toulouse-Purpan, F-31300 Toulouse, France; severine.fruchon@inserm.fr (S.F.); nicolas.beton@inserm.fr (N.B.); cecile.goursat@inserm.fr (C.G.); 2 CNRS, UMR 5089, Université de Toulouse, UPS, Institut de Pharmacologie et de Biologie Structurale, IPBS, 205 route de Narbonne, BP 64182, F-31077 Toulouse CEDEX 4, France; elisabeth.bellard@ipbs.fr (E.B.); muriel.golzio@ipbs.fr (M.G.); 3 CNRS, UPR 8241, Laboratoire de Chimie de Coordination, 205 route de Narbonne, BP 44099, F-31077 Toulouse CEDEX 4, France; abdelouahd.oukhrib@lcc-toulouse.fr (A.O.); anne-marie.caminade@lcc-toulouse.fr (A.-M.C.); cedric-olivier.turrin@lcc-toulouse.fr (C.-O.T.); 4 LCC-CNRS, Université de Toulouse, CNRS, Toulouse, France; 5 CNRS, UMR 5623, Université de Toulouse, UPS, Laboratoire des Interactions Moléculaires et Réactivité Chimique et Photochimique, IMRCP, 118 route de Narbonne, F-31062 Toulouse CEDEX 9, France; blanzat@chimie.ups-tlse.fr

**Keywords:** azabisphosphonate, biodistribution, dendrimers, drug candidate, preclinical development, rabbit, rodents, safety

## Abstract

Dendrimers are nanosized, arborescent polymers of which size and structure are perfectly controlled. This is one reason why they are widely used for biomedical purposes. Previously, we showed that a phosphorus-based dendrimer capped with anionic azabisphosphonate groups (so-called ABP dendrimer) has immuno-modulatory and anti-inflammatory properties towards human immune cells in vitro. Thereafter, we have shown that the ABP dendrimer has a promising therapeutic efficacy to treat models of chronic inflammatory disorders. On the way to clinical translation, the biodistribution and the safety of this drug-candidate has to be thoroughly assessed. In this article, we present preliminary non-clinical data regarding biodistribution, hematological safety, genotoxicity, maximal tolerated doses, and early cardiac safety of the ABP dendrimer. One of the genotoxicity assays reveals a potential mutagen effect of the item at a concentration above 200 µM, i.e., up to 100 times the active dose in vitro on human immune cells. However, as the results obtained for all the other assays show that the ABP dendrimer has promising biodistribution and safety profiles, there is no red flag raised to hamper the regulatory pre-clinical development of the ABP dendrimer.

## 1. Introduction

**Figure 1:**
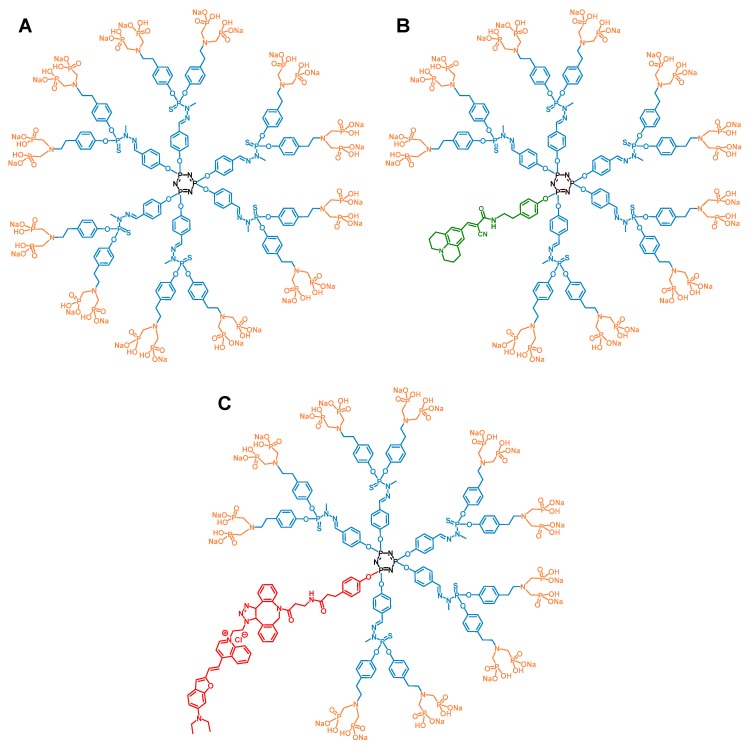
Structure of the three dendrimers used in this study. The cyclotriphosphazene core (N_3_P_3_) is in black, the poly(phosphorhydrazone) (PPH) branches (including the point of divergence) are in blue. The tyramine-based (in blue) azabisphosphonate (ABP) surface groups are in orange. (**A**) The ABP dendrimer. (**B**) The ABP-Julolidine dendrimer. (**C**) The ABP-near infrared reflectance (NIR) dendrimer.

Dendrimers are nanosized non-linear polymers synthesized through a stepwise iterative process. Therefore, their size and structure are perfectly defined, and their controlled synthesis affords consistent batches of monodisperse compounds. Some of their intrinsic features such as their supramolecular properties, their size and globular shape, and their multivalency make them attractive nanomolecules for biological and medical applications [1]. Dendrimers can be divergently built on a central core on which a first series of radial branches ending by a point of divergence is linked. The latter enables the dendritic growth of the molecule by addition of supplemental branches, if any. Finally, the synthesis ends with the addition of functional groups at the outermost series of branches. The number of series of branches determines the generation of the dendrimer. It is generally assumed that the size of a first-generation dendrimer begins at 1–2 nm, and that an additional 1 nm is gained linearly with each supplemental generation, as determined by size-exclusion chromatography [2], and electron microscopy [3]. Several families of dendrimers have shown immunomodulatory properties towards the human immune system [1]. Anti-inflammatory activation of the immune system is searched to control inflammatory disorders. Previously, we have shown that a first generation (G1) phosphorus-based dendrimer capped with anionic azabisphosphonate groups (so-called ABP dendrimer, Figure 1A) borne by poly(phosphorhydrazone) (PPH) branches has immuno-modulatory and anti-inflammatory properties towards different subpopulations of the human immune system [4,5,6,7].

We have also shown that the ABP dendrimer dramatically inhibits the onset and development of experimental arthritis in mouse models relevant to human rheumatoid arthritis, a chronic inflammatory disease of auto-immune origin [8,9]. The ABP dendrimer has also proven to be efficient in a rat model of uveitis [10], and in a mouse model of experimental autoimmune encephalomyelitis relevant to human multiple sclerosis [11]. These studies make the case for the ABP dendrimer as an innovative drug-candidate for the treatment of inflammatory disorders [12]. Nevertheless, the development of new medicines is a challenging issue as the therapeutic benefit of a drug-candidate should not be depreciated by a poor tolerance and deleterious side effects. This is the benefit/risk ratio. In particular, nanosized molecules intended for human use raise the issue of their toxicity [13,14] and immuno-safety [15]. That is why a couple of years ago, we have assessed the biological risk of the ABP dendrimer in a non-human primate model, much more relevant to humans than classical rodent models [16]. Along repeated intravenous injections in cynomolgus monkeys (*Macaca fascicularis*), we have shown that the ABP dendrimer has no deleterious effects. Numerous biochemical, hematological, clotting, and immunological parameters have been assessed and are kept in the physiological ranges during the duration of the study. Quantification of serum cytokines and histopathological analyses after necropsy failed to reveal any noticeable lesion or noteworthy non-physiological occurrence. However, despite their not questionable relevance, non-human primate models are not included in the range of regulatory models to assess the preclinical safety of drug-candidates. The reasons lie in cost and ethics issues. Therefore, in this new study we have assessed the preclinical biodistribution and safety of the ABP dendrimer in rodents: Whole body biodistribution and hematological safety in mice, in vitro genotoxicity in bacteria and mammalian cells, maximal tolerated dose (MTD) in mice and rats, and in vitro cardiac safety.

## 2. Materials and Methods

### 2.1. Dendrimers

The three dendrimers used in this study have been synthesized at the Laboratoire de Chimie de Coordination, Toulouse, France. The synthesis of the ABP dendrimer and of its green fluorescent analogue ABP-Julolidine have been already described [17,18]. The near infrared fluorescent analogue ABP-NIR was afforded by the “Laboratoire de Chimie de Coordination” for this study (A. Oukhrib, A.M. Caminade, C.O. Turrin).

#### Zeta Potential of the Dendrimers

Zeta potential measurements were performed on a Nano ZS instrument (Malvern Panalytical Ltd, Malvern, UK), with a He–Ne laser (633 nm), using an electrophoretic light scattering technique. The analyses were done at 25.0 ± 0.1 °C with dendrimer solutions at 100 µM in Milli-Q water (Merck KGaA, Darmstadt, Germany), and analyzed with the Smoluchowski model.

### 2.2. Biodistribution

Studies in animals were conducted in accordance with the principles and procedures outlined by the European convention for the protection of vertebrate animals used for experimentation and with approval from the Local Ethical Review Committee (n°20090202/51). The mice were housed in groups of five in plastic cages in an air-conditioned room (temperature of 22 ± 2 °C and relative humidity between 30% and 50%) with 12 h of artificial light/12 h dark. Food and drinking water were provided ad libitum.

#### 2.2.1. Biodistribution Using the ABP-Julolidine Dendrimer

**Figure 2:**
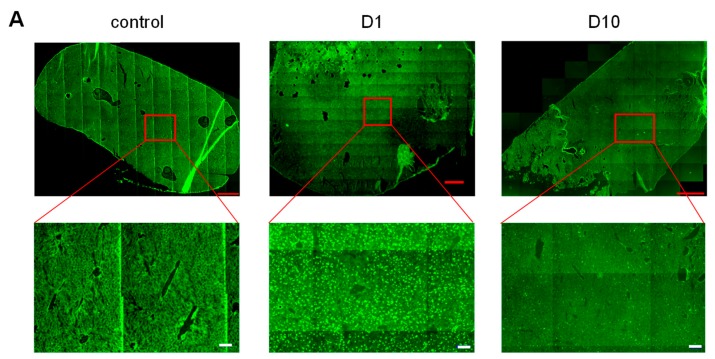

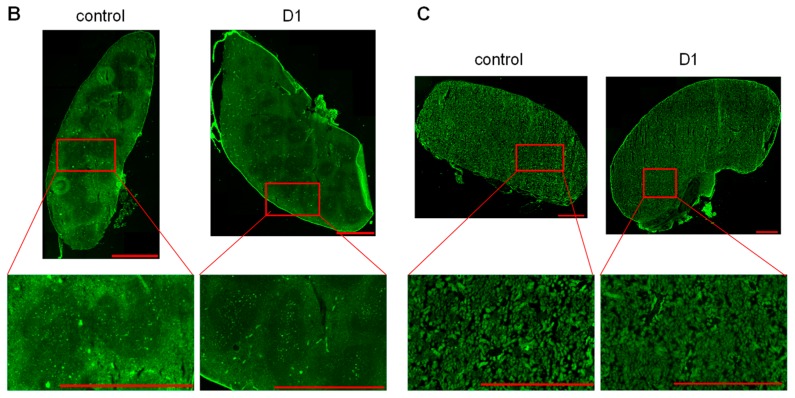
Fluorescence images of entire organs after an intravenous (IV) injection of the green fluorescent ABP-Julolidine dendrimer. Control mice have been IV injected with phosphate buffer saline (PBS). (**A**) Liver at days one and 10. (**B**) Spleen at day one. (**C**) Kidneys at day one. Red bars and white bars (for the zoom on the liver) represent 1 and 0.1 mm, respectively. Images are representative of one mice out of six.

**Figure 3:**
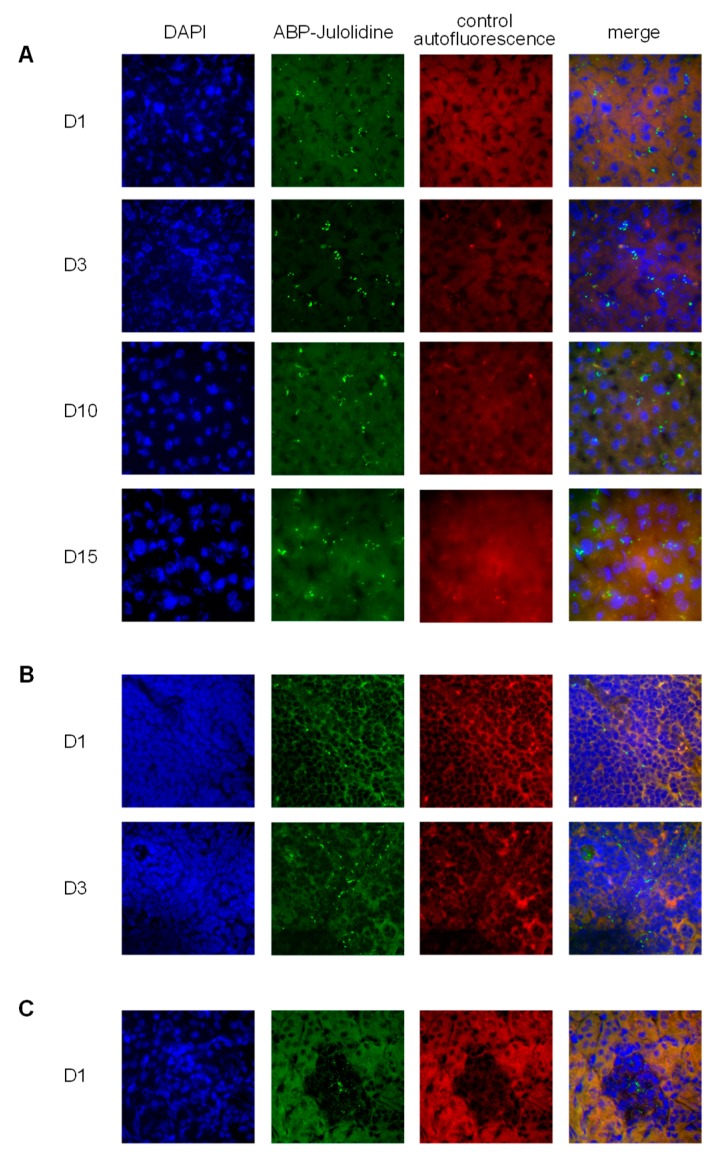
Fluorescence images at the sub-organ level after an IV injection of the ABP-Julolidine dendrimer (in green, second column), with DAPI (4′,6-diamidino-2-phénylindole) nuclear staining (in blue, left column; merge on the right column), and the auto-fluorescence of the tissues as internal controls (in red, third column) for the liver at days one, three, 10 and 15 (**A**), the spleen at days one and three (**B**), and the kidney at day one (**C**). Images are representative of one mice out of six.

Female hairless SKH1 mice were obtained from Charles River Laboratories (Saint-Germain-Nuelles, Saint-Germain-Nuelles, France). The ABP-Julolidine dendrimer was diluted in a phosphate buffer saline (PBS) at a concentration of 0.5 mM. The injected volume (less than 100 µL) was adapted to the weight of the animal for a final dose of 10 mg/kg. The volume was injected through intraperitoneal (IP), subcutaneous (SC), and intravenous (IV, in the tail) routes. Control mice were injected with PBS alone. At different time points (day one, three, 10 and 15 after injection), mice were sacrificed and specific organs (liver, kidneys, lungs, spleen, and lymph nodes) were collected. Organs were immediately embedded in optimal cutting-temperature compounds (isopentane) and snap-frozen on liquid nitrogen. 7 µm thick cryo-sections were cut, fixed for 10 min in cooled acetone, washed in PBS, counterstained with the nuclear dye DAPI, and mounted in a Mowiol medium. For the whole organ analysis, slides were observed on a fluorescence-inverted wild field microscope Apotome (Zeiss, Oberkochen, Germany) equipped with a 40X objective, and suitable filters for the detection of the ABP-Julolidine dendrimer. The λ_exc_ range was 450–490 nm, and the λ_em_ range was 515–560 nm. Images were analyzed using the ZEN Software (Figure 2). For the sub-organ analysis, slides were observed on a fluorescence-inverted microscope DMIRB (Leica, Wetzlar, Germany) equipped with a 40X objective, and suitable filters for the detection of DAPI and of the ABP-Julolidine dendrimer. The λ_exc_ ranges were 340–380 nm and 460–500 nm, and the λ_em_ ranges were above 425 nm and 512–542 nm, respectively. Images were analyzed using the Metamorph Software (Molecular Devices, San Jose, CA, USA) (Figure 3). 

#### 2.2.2. Biodistribution Using the ABP-NIR Dendrimer

**Figure 4:**
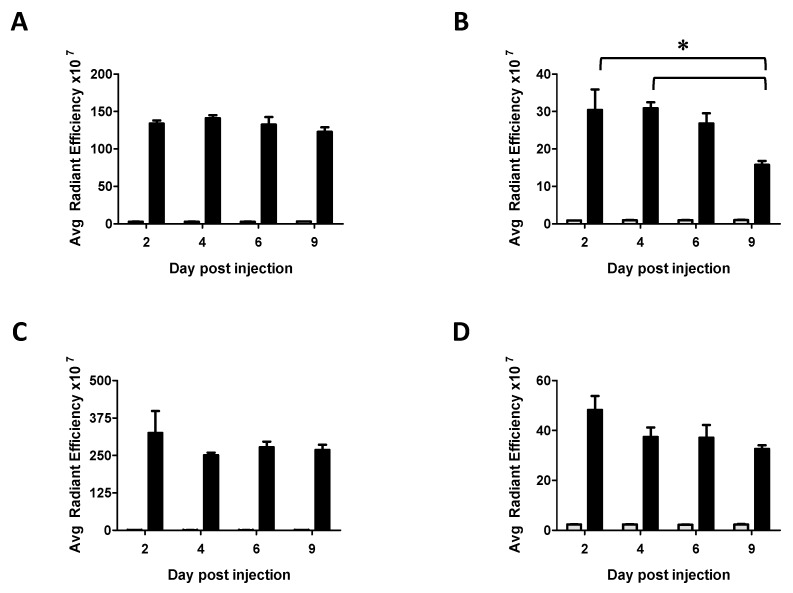
Quantification of the average fluorescence per square centimeter of isolated organs after one IV injection of the ABP-NIR dendrimer using the IVIS (In Vivo Imaging System) Spectrum imaging system. (**A**) Liver. (**B**) Spleen. (**C**) Lungs. (**D**) Kidneys. Grey bars are control mice IV injected with PBS (*n* = 3 mice at each time point), black bars are mice IV injected with the ABP-NIR dendrimer (*n* = 5 mice at each time point). * *p* < 0.05 using One Way ANOVA or Kruskal-Wallis’ (for the lungs) tests, followed by post hoc Bonferroni’s and Dunn’s tests, respectively.

**Figure 5:**
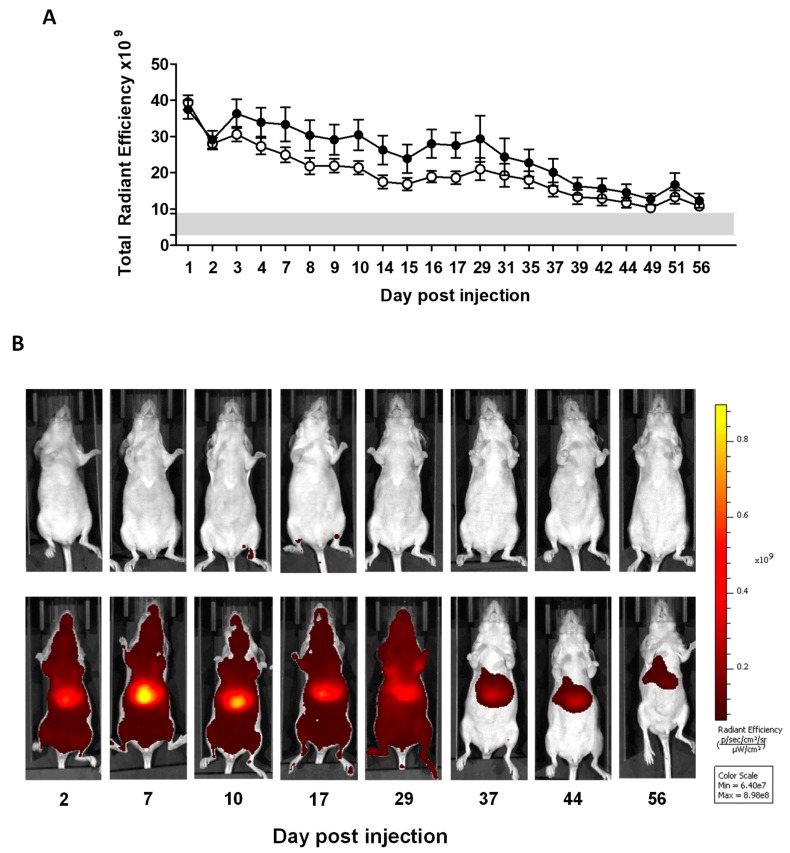
(**A**) Follow-up of the fluorescence in living mice after one IV injection of the ABP-NIR dendrimer. Whole body fluorescence was measured using the IVIS Spectrum imaging system. Black dots and white dots represent the values obtained on the ventral and on the dorsal sides, respectively (mean ± SEM, *n* = 6 mice). The grey bar represents the mean ± 2 SD of the fluorescence values measured for the dorsal side of the control group mice IV injected with PBS (*n* = 2 mice). (**B**) Images from the ventral side of one mouse representative of each group. The intense fluorescent spot encompasses the abdominal organs.

Female C57BL/6J and athymic nude mice (eight weeks of age), for ex vivo and in vivo imaging respectively, were purchased from Envigo (Gannat, France). Fluorescence monitoring and quantification were performed after an IV injection of the ABP-NIR dendrimer at 50 mg/kg in a volume of 100 µL into the tail vein. The control mice were injected with 100μl of PBS. For the ex vivo quantification of fluorescence, mice were sacrificed at different time points (day two, four, six and nine after injection), and specific organs (liver, kidneys, lungs, and spleen) were collected and maintained on ice until the quantification of fluorescence. For the in vivo quantification of fluorescence, a longitudinal monitoring has been performed for 56 days on whole animals under gas anesthesia with 2.5% isoflurane. For both experiments, images were acquired with the IVIS Spectrum imaging system (PerkinElmer, Waltham, MA, USA) with the 640 nm excitation and the 780 nm emission filters, epi-illumination, and auto settings for exposure parameters. Fluorescence intensities were quantified on the images with the Living Image 4.5 software (PerkinElmer, Waltham, MA, USA). The region of interest (ROI) were drawn around the isolated organs or the whole body to measure the fluorescence intensity, and data are expressed as the average radiant efficiency in [photon/s/cm^2^/sr]/[µW/cm^2^] for the isolated organs (Figure 4), and as the total radiant efficiency as [photon/s]/[µW/cm^2^] for the whole bodies (Figure 5).

### 2.3. Hematological Safety

Female hairless SKH1 mice were obtained from Charles River Laboratories. The ABP dendrimer was diluted in PBS at a concentration of 0.5 mM. The injected volume (less than 100 µL) was adapted to the weight of the animal for a final dose of 10 mg/kg. Control untreated mice were injected with the corresponding volume of PBS. Blood samples were obtained from retro-orbital puncture and were collected in EDTA tubes 24 h after an IV injection in the tail. Hematologic parameters were measured on 20 µL of blood samples with the ABX Micros 60 analyzer (Horiba ABX SAS, Montpellier, France). Absolute numbers of red blood cells (RBC) were determined, and hemoglobin (HGB, g/dL), hematocrit (HCT, %), mean corpuscular volume (MCV, µm^3^), mean corpuscular hemoglobin concentration (MCHC, g/dL), mean corpuscular hemoglobin (MCH, pg), and RBC distribution width (RDW, %) were quantified. Absolute numbers of platelets (PLT), and mean platelet volume (MPV, µm^3^) were determined. Finally, absolute numbers of white blood cells (WBC) were determined. Among WBC, the absolute numbers and percentages of lymphocytes (LYC), monocytes (MON), and granulocytes (GRA) were assessed. Finally, the enzymatic activities of the aspartate transaminase (AST) and alanine transaminase (ALT) were measured in plasma with the Cobas Mira Plus Chemistry Analyzer (Horiba ABX SAS, Montpellier, France).

### 2.4. Genotoxicity

The studies were performed at the Pasteur Institute, Lille, France.

#### 2.4.1. BN Ames’ Test

The standardized test is based on three different mutated strains of *Salmonella typhimurium* that are auxotroph for histidine (TA 1537, TA 100, TA 98). The potential genotoxicity of tested compounds is assessed on each strain with (for the detection of pro-mutagen activity) and without (for the detection of mutagen activity) metabolic activation by a microsomal fraction of rat liver (S9 mix, see below). Genotoxicity is assessed by quantifying the number of reverse mutations leading to *S. typhimurium* that are prototroph for histidine (i.e., able to grow on a histidine-free medium). The experimental procedure has been accurately described [19]. Briefly, *S. typhimurium* strains have been pre-incubated with the ABP dendrimer in 96-well plates. Nine successive two-fold dilutions of the ABP dendrimer were prepared directly in the 96-well plate starting with 20 µL of the initial solution at 10 mg/mL, i.e., 200 µg of the ABP dendrimer. Therefore, the lowest dose was 0.39 µg of the ABP dendrimer (Table 1). Then 100 µL of the bacterium suspension and 80 µL of PBS were added (final volume of 200 µL/well). After 90 min at 37 °C with gentle shaking, the total volume of each well was added to a 2 mL solution of soft agar in a tube, which was finally spread on solid agar in a dish. Dishes are incubated at 37 °C for 48 h, and the number of colonies was counted in each dish. Negative (no ABP dendrimer) and positive controls (see Table 1) are implemented in the same 96-well plate. Each condition was performed in triplicate.

A complementary assay on the strain TA98 without metabolic activation has involved higher doses. To reach the maximal dose of 1000 µg/dish, 100 µL of the initial solution at 10 mg/mL was added in each well (replacing the PBS) for the pre-incubation test. Four successive two-fold dilution enabled doses of 500, 250, 125, and 62.5 µg/dish. 

The S9 microsomal fraction of rat liver was obtained as already described [19] (pp. 186–187). The S9 fraction was stored in liquid nitrogen. The S9 mix is prepared extemporaneously by adding 1 mL of KCl 150 mM, 1 mL of Glucose-6-phosphate 1 M, and 1 mL of NADP^+^ 34 mM to a thawed 2 mL aliquot of the S9 [20].

#### 2.4.2. In Vitro Micronucleus Test

L5178Y cells in the exponential growth phase were treated in 96-well plates with different concentrations of the ABP dendrimer, with (for the detection of pro-mutagen activity) and without (for the detection of mutagen activity) metabolic activation by a microsomal fraction of rat liver (S9 mix, see above). L5178Y cells were cultured in RPMI 1640 complemented with 10% of heat-inactivated horse serum (FM10 medium). Three different treatments were implemented [21]: A 4 h treatment with metabolic activation followed by a recovery period of 24 h (T1); two 24 h treatments without metabolic activation, one with no recovery period (T2), one followed by a recovery period of 20 h (T3). In parallel, a cytotoxicity assay is performed on the cells using the classical colorimetric assay based on the enzymatic reduction of the tetrazolium dye MTT to its insoluble formazan [22,23], in the same conditions (concentrations of the ABP dendrimer, and metabolic activation or not). 

Ten successive two-fold dilutions of the ABP dendrimer were prepared directly in the 96-well plate starting with 50 µL (cytotoxicity) or 100 µL (micronucleus test) of the initial solution at 10 mg/mL, i.e., 1.72 mM, of ABP dendrimer in FM10. Therefore, the highest concentration is 859 µM, and the lowest is 0.839 µM. Of note, in the assay with metabolic activation, the addition of 10% of the S9 mix consequently lowered the concentrations of the ABP dendrimer to 773 µM for the highest one, and 0.755 µM for the lowest one. Then 50 µL (cytotoxicity, final volume 100 µL) or 100 µL (micronucleus test, final volume 200 µL) of L5178Y cell suspension were added in each well.

The MTT assay was used to select the doses of the ABP dendrimer for which the micronucleus test has been analyzed. For each condition, the percentage of relative survival was calculated as the ratio (absorbance of the condition)/(absorbance of the untreated control) × 100. For each treatment, three successive concentrations of the ABP dendrimer were selected. The criteria were the following: The highest concentration had a percentage of relative survival above 50%, and the lowest concentration of the series had a percentage of relative survival above 75%. Therefore, for T1 the concentrations were 773, 387, and 193 µM; for T2 the concentrations were 215, 107, and 53.7 µM; for T3 the concentrations were 430, 215, and 107 µM.

Then, the 96-well plates for the micronucleus test were centrifuged, the supernatant was flushed, and the cells were washed once with 150 µL of the FM10 medium. The cells in the wells selected for the micronucleus test according to the MTT test were incubated with 150 µL of a hypotonic medium for 4 min at room temperature, centrifuged, and the supernatant was replaced by 150 µL of the fixation medium (ethanol/acetic acid:3/1) for 10 min at room temperature. Then, three drops of the suspension were deposited on a slide and dried for 24 h in ambient air. Finally, cells were stained during 10 min in a water solution with 2% Giemsa. After intensive washing with water, 2000 cells were analyzed under a microscope and micronucleus were counted. To be considered as a micronucleus, an intra-cellular structure had to have a color less intense than that of the main nucleus of the cell, its diameter had to be less than one third of the area of the main nucleus, it had to be clearly circled by a nuclear membrane, and it had no contact nor was linked to the main nucleus by a nucleoplasmic bridge [24]. Finally, to exclude apoptosis and DNA fragmentation, cells with more than five micronuclei were excluded from the counting.

### 2.5. Maximal Tolerated Dose (MTD)

The study in mice was performed at the Pasteur Institute, Lille, France (project identification code FSR-IPL-04070-1), and the studies in rats were performed at Ricerca Biosciences SAS, Saint-Germain/l’Arbresle, France (project identification code AA96166), in accordance with the European Convention and with the Institutional Animal Care.

#### 2.5.1. Single Injection in Mice and Rats

Studies were performed in OF1 mice and Wistar rats (approximately six weeks of age), in both males and females. Animals were housed in groups of three for the mice, of two for the rats, of the same sex and dose group in plastic cages in an air-conditioned room (temperature of 22 ± 3 °C and relative humidity of 55% ± 15%) with 12 h of artificial light/12 h dark. Food and drinking water were provided ad libitum. The ABP dendrimer was prepared in NaCl 0.9% just before an IV injection using a microflex infusion set introduced into the tail vein. The administered volume was 5 mL/kg for mice and 2 mL/kg for rats. The dose volumes were calculated using the latest recorded body weight of the animals. In mice, the dose range was 50, 100, 150, and 250 mg/kg. Mice were observed during 15 min after dosing, then 1 h, 2 h, 6 h, and daily during 14 days to detect any clinical signs or reactions to treatment. Animals were weighted at days zero (before the dosing), two, seven and 14. In rats, the dose range was 50, 100, and 200 mg/kg. Rats were observed after the dosing and euthanized two days after the dosing. 

#### 2.5.2. Repeated Injections in Rats

The study was performed in Wistar rats (approximately six weeks of age), in both males and females. Housing and diet conditions were the same than in the single administration study (see above). The dose range was 30, 60, and 120 mg/kg/day during seven consecutive days. Rats were observed before and at least once after the dosing. Animals were weighted at days zero (before the first administration) and seven (before euthanasia).

### 2.6. In Vitro Cardiac Safety

These experiments were performed at Physiostim, Lautrec, France.

Female white New-Zeland rabbits were obtained from Charles River Laboratories. Six Purkinje fibers were obtained from left ventricles of six animals anaesthetized with sodium pentobarbital (0.9 to 1.0 mL/kg, IV) and were maintained at 36 ± 0.5 °C in a 5 mL bath. The ABP dendrimer was dissolved in distilled water (DW, vehicle) at 5 mM. Experiments were performed on two groups of three fibers (control group of the DW vehicle, group treated with the ABP dendrimer). In a first experiment, each fiber was electrically stimulated at 1 Hz, then superfused with Tyrode’s solution (prevalue), DW 0.1% (baseline). In the ABP dendrimer treated group, the three fibers were then superfused with increasing concentrations of the ABP dendrimer (0.05, 0.5, and 5 µM) for 30 min followed by Quinidine 10 µM for 20 min. In the DW vehicle group, the three fibers were then superfused with several periods of DW at 0.1% (DW1, DW2, and DW3) for 30 min followed by Quinidine at 10 µM for 20 min. Quinidine is a positive control indicating that, at the end of the experimental sequence, the fibers are responsive to an anti-arrhythmic drug that increases the action potential of the fiber and modifies some of the action potential parameters (see below).

In a second experiment, the same fibers underwent an electric stimulation at 0.2 Hz for at least 3 min followed by 5 min recovery to 1 Hz, then superfused with the same solutions and the same sequence than during the first experiment.

Action potential parameters that were measured are: Resting potential (RP), action potential durations at 50%, 70% and 90% of repolarization (APD_50_, APD_70_ and APD_90_, respectively), action potential amplitude (mV), and maximal rate of depolarization dV/dt_max_ (V/s).

### 2.7. Statistical Analyses

All statistical analyses have been performed with Prism 5.0 (GraphPad, San Diego, CA, USA).

#### 2.7.1. Biodistribution Using the ABP-NIR Dendrimer

In Figure 4, following the ex vivo quantification of fluorescence in each isolated organ, the homogeneity of the variances at the different time points was analyzed using the Bartlett’s parametric test. When the homogeneity of the variances was verified (for the liver, the spleen, and the kidneys), a One Way ANOVA test was used to detect differences between the different time points for each isolated organ. When a difference was detected (spleen), the post-hoc Bonferroni’s test was performed to evidence a statistically significant difference. For the lungs, the homogeneity of variances was not verified. Therefore, we applied the non-parametric Kruskal-Wallis’ test followed by the post-hoc Dunn’s test.

#### 2.7.2. Hematological Safety

**Figure 6:**
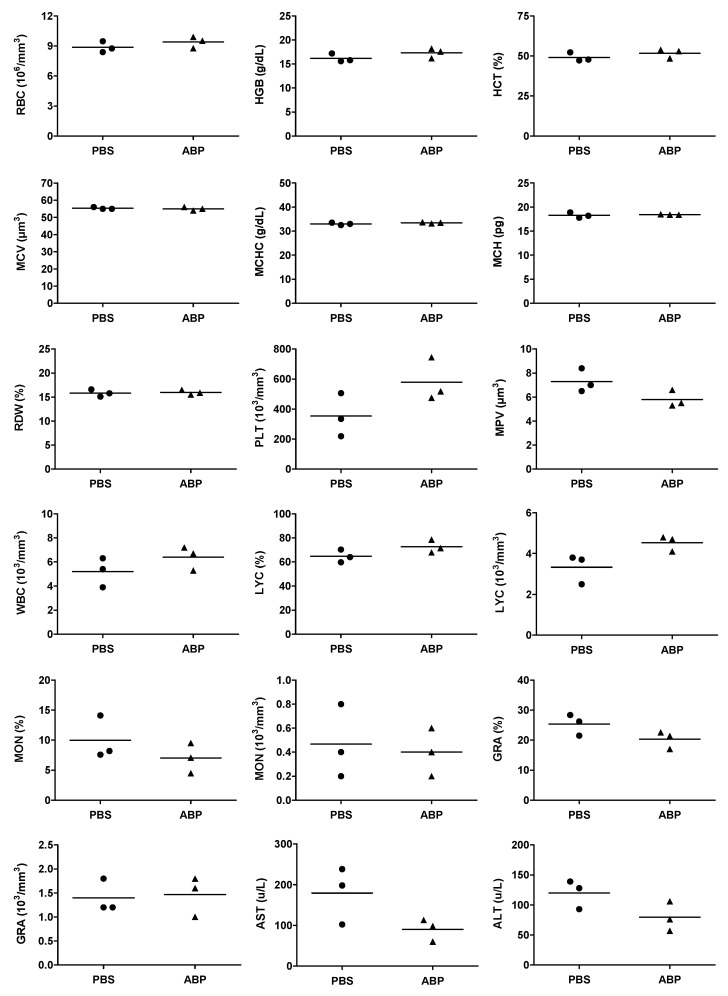
Quantification of the absolute numbers of red blood cells (RBC), hemoglobin (HGB), hematocrit (HCT), mean corpuscular volume (MCV), mean corpuscular hemoglobin concentration (MCHC), mean corpuscular hemoglobin (MCH), RBC distribution width (RDW), absolute numbers of platelets (PLT), mean platelet volume (MPV), absolute numbers of white blood cells (WBC), absolute numbers and percentages of lymphocytes (LYC), monocytes (MON), and granulocytes (GRA) in blood; quantification of the enzymatic activities of aspartate and alanine transaminases (AST and ALT) in plasma. Measurements have been performed 24 h after an IV injection of the ABP dendrimer at 10 mg/kg. Bars represent the mean of each group of *n* = 3 mice.

In Figure 6, to compare the quantification of the different hematological parameters between the ABP dendrimer treated and the control untreated mice, the homogeneity of the variances was analyzed using the Fisher’s test. When the homogeneity of the variances was verified (all parameters except the MCH), a Student’s t-test was performed. For the MCH, as the variances were not homogeneous, the unequal variances Welch’s t-test was applied.

#### 2.7.3. Genotoxicity

For the BN Ames’ test, the post-hoc Dunett’s test was used after a One Way ANOVA to compare the multiple ratio of mutants per dish. For the micronucleus test, the chi-squared test was used to determine whether there was a significant difference between the observed frequencies of micronuclei at the different concentrations of the ABP dendrimer versus untreated cells.

## 3. Results

### 3.1. Biodistribution

In preclinical mouse models of chronic inflammatory disorders, we have proven the therapeutic efficacy of the ABP dendrimer through IV injections [8,11]. This systemic route of administration urges the questions of the biodistribution of the molecule in the body of administered animals and of its potential accumulation therein. In a first series of experiments, we have taken advantage of an already available fluorescent analogue of the ABP dendrimer, which emits green fluorescence thanks to the presence of a Julolidine group in place of one of the six branches of the ABP dendrimer (the so-called ABP-Julolidine dendrimer, Figure 1B) [18]. The ABP and ABP-Julolidine dendrimers have close molecular weights (5820 and 5259 g/mol, respectively) and zeta potentials (−51 ± 8 and −55 ± 7 mV, respectively). Moreover, we have shown that the ABP-Julolidine dendrimer is as active as the ABP dendrimer, both in vitro on human monocytes [18] and in vivo in a mouse model of experimental arthritis [9]. Therefore, it can be assumed that the biodistribution of the ABP-Julolidine dendrimer is relevant to that of the ABP dendrimer. Several systemic routes of administration have been assessed with a unique injection at the therapeutic active dose of 10 mg/kg [8,9,11]. Using the IP and SC routes, we have shown that the fluorescent dendrimer stayed at the site of injection for several days and is not distributed (data not shown). On the contrary, using an IV injection in the tail, the ABP-Julolidine dendrimer can be detected by fluorescence microscopy in draining lymph nodes as expected (not shown). In the liver at day one, the green aggregates that can be observed in the ABP-Julolidine treated mice and which are not seen in the control mice show the presence of the molecule (Figure 2A). At the same time point, the fluorescence images of the spleen and the kidneys were the same than the ones obtained from non-treated mice (Figure 2B,C). At day 10, the quantity of the ABP-Julolidine dendrimer in the liver seems to have dramatically decreased (Figure 2A).

To better appreciate the biodistribution of the ABP-Julolidine dendrimer in these organs, we have imaged co-staining of the ABP-Julolidine dendrimer and DAPI, a nuclear fluorescent probe. The auto-fluorescence of the tissues has been imaged as the control. We confirm that the dendrimer is mainly found in the liver until day 15 (Figure 3A). It is also detected in the spleen until day three (Figure 3B), and in very little proportion in the kidneys at day one (Figure 3C). For the spleen and the kidneys, images after day three and day one, respectively, are not shown because the ABP-Julolidine dendrimer is no more visible.

However, as it can be seen on the images of Figure 2 and Figure 3, the auto-fluorescence of the tissues causes a green background that is a strong limitation for quantifying the fluorescence of the ABP-Julolidine dendrimer. Therefore, we have designed and synthesized a new analogue of the ABP dendrimer which emits near infrared fluorescence, not overlapping auto-fluorescence of the tissues (the so-called ABP-NIR dendrimer, Figure 1C, A. Oukhrib, A.M. Caminade, and C.O. Turrin personal data). The ABP-NIR dendrimer has a molecular weight (5746 g/mol) and a zeta potential (−55 ± 5 mV) close to the ones of the ABP and ABP-Julolidine dendrimers. Moreover, we have checked that the ABP-NIR dendrimer has the same biological properties than the ABP dendrimer in vitro (S. Fruchon and R. Poupot personal data). Therefore, it can be assumed that the biodistribution of the ABP-NIR dendrimer is the same than that of the two other dendrimers. Mice have been IV injected with the ABP-NIR dendrimer at 10 mg/kg. At different time points, the liver, the spleen, the lungs, and the kidneys have been isolated and their average fluorescence per square centimeter has been quantified using the IVIS Spectrum imaging system (Figure 4). The ABP-NIR dendrimer is mainly found in the lungs (Figure 4C), and in the liver (Figure 4A). Lower quantities are detected in the spleen and in the kidney. Regarding the kinetics of the biodistribution, the only statistically significant decreases are observed in the spleen between days two and nine, and between days four and nine. The downward trend seen in the kidneys is not statistically significant at day nine.

Finally, to assess a potential bio-accumulation of the ABP-NIR dendrimer, we have quantified the total amount of fluorescence in living animals during a follow-up of 56 days using the IVIS Spectrum imaging system. Both the ventral and the dorsal sides of the animals have been analyzed, and the quantification is compared to the one of non-treated mice (Figure 5). The curves are visually fitting with a linear decrease of the fluorescence of the ABP-NIR dendrimer in the animals, due to its progressive elimination. At the end of the 56 day follow-up, the fluorescence of the injected animals almost reaches the one of non-treated controls.

### 3.2. Hematological Safety

As the ABP dendrimer is a potent immuno-modulatory compound [12], its potential effect on cellular components of the peripheral blood of mice has been assessed. We have quantified the red blood cells (RBC) and the usual parameters related to these cells. We have also quantified the number and volume of platelets, and the number of white blood cells (WBC) together with the numbers and percentages of lymphocytes (LYC), monocytes (MON), and granulocytes (GRA) among them. Finally, the enzymatic activities of aspartate and alanine transaminases (AST and ALT) have been measured. As shown in Figure 6, none of these 18 blood parameters are changed after an IV injection of the ABP dendrimer.

### 3.3. Genotoxicity

The testing of genotoxicity is a pivotal step in the assessment of the safety of all types of substances intended for human and animal uses, especially drugs [25]. Indeed, genotoxicity is a red flag in drug development as it can increase the susceptibility of treated patients to cancers and genetic diseases. Several in vitro and in vivo assays exist to assess genotoxicity. In early steps of drug development, in vitro tests are generally preferred as they are more rapid, cheaper, they require less amount of molecule, and they are considered predictive and reliable. The two most recognized tests in drug development are the BN Ames’ test [26] and the micronucleus test [27]. We have used both of them to evaluate the genotoxicity of the ABP dendrimer.

#### 3.3.1. BN Ames’ Test

In this test, mutagen products are evidenced by the reverse mutations that they induced in the *his^-^* locus of *Salmonella typhimurium* strains, which are auxotroph for histidine. The test has been standardized [19], and the micromethod test we have chosen uses three different strains of *S. typhimurium*: TA 1537, TA 98, and TA 100 that enable to highlight the frameshift mutations by deletion and by insertion, and the substitution of base pairs, respectively. Moreover, the implementation of the test without metabolic activation reveals direct mutagenic compounds, whereas the implementation of the test with metabolic activation reveals pro-mutagen ones. In the experimental conditions that have been used, and based on the historical experience of the laboratory (Pasteur Institute, Lille, France), the criteria to make the decision of the genotoxicity of a compound are the following:

- for the strain TA 1537, a compound that induces a dose-dependent increase in the number of reverse mutations along at least three consecutive doses, with the greatest increase being greater than or equal to three times that of the untreated control (which gives the number of spontaneous reverse mutations) is considered positive to the test;
- for the strains TA 100 and TA 98, a compound that induces a dose-dependent increase in the number of reverse mutations along at least three consecutive doses, with the greatest increase being greater than or equal to twice that of the untreated control (which gives the number of spontaneous reverse mutations) is considered positive to the test.

The test has been performed with ten doses of the ABP dendrimer ranging from 0.39 to 200 µg/dish. The results are presented in Table 1. The statistical analysis thereof evidences an increase in the number of reverse mutations in the strain TA 100 with metabolic activation at the dose of 6.25 µg of the ABP dendrimer per dish, and another one in the strain TA 98 without metabolic activation, at the dose of 200 µg per dish. The former increase only appears at the dose of 6.25 µg/dish and is less than twice the number of spontaneous reverse mutations in the untreated control. Although the second increase was also lower than twice the number of spontaneous reverse mutations in the untreated control, we have implemented an independent complementary assay with higher doses (62.5, 125, 250, 500 and 1000 µg/dish) to see whether this increase was also observed at doses higher than 200 µg/dish (which was the highest dose tested in the first assay) to fulfill the dose-dependent criterion. In this additional assay, there was no significant increase in the number of reverse mutations induced by the ABP dendrimer, the highest ratio being 1.2 at the dose of 500 µg/dish (data not shown). Therefore, the ABP dendrimer is not considered as genotoxic according to the BN Ames’ test.

#### 3.3.2. In Vitro Micronucleus Test

This test enables the simultaneous detection of mitotic delay, apoptosis, chromosome breakage, chromosome loss, and non-disjunction. Each of these genetic abnormalities results in the formation of micronuclei in vitro in cells of mouse lymphoma L5187Y. The cells having more than five micronuclei in their cytoplasm are counted under a microscope. In order to avoid the counting of micronuclei appearing after apoptosis induced by the cytotoxicity of the tested item, a MTT viability test is performed in parallel with the micronucleus test itself. At the end, cells with micronuclei are counted only in the three highest consecutive non-cytotoxic concentrations of the compound. In the experimental conditions that have been used, a compound is considered as genotoxic if it induces a statistically significant increase in the number of cells with micronuclei when compared to the untreated control, the number of these cells being at least twice that of the untreated control, and providing there is a concentration-dependent effect.

The MTT and the micronucleus tests have been performed with eleven concentrations of the ABP dendrimer ranging from 0.839 to 859 µM. The counting of the cells with micronuclei for the three concentrations selected from the MTT test for each condition are presented in Table 2. The statistical analysis thereof evidences an increase in the number of cells with micronuclei in L5187Y cells when the test has been performed without metabolic activation and with a recovery period after incubation with the ABP dendrimer. Indeed, the number of cells with micronuclei was 12 at 215 µM and 17 at 430 µM, whereas it was four in the untreated control. Hence, there is a concentration-dependent effect and the numbers are more than twice that of the untreated control. Therefore, the ABP dendrimer is considered as potentially genotoxic according to the micronucleus test at the concentration of 215 µM.

### 3.4. Maximal Tolerated Dose (MTD)

The MTD is the highest dose of a substance with no visible adverse clinical effect on the living organism to which it is administered. Adverse clinical effects may include unsteady gait, bristly hairs, cries when touched, decreased activity (including food uptake), breathing difficulties, rotations in the cage.

#### 3.4.1. Single Injection in Mice and Rats

The aim was to assess the MTD following a single intravenous administration of the ABP dendrimer. At first, the assay was performed in mice. Four doses (50, 100, 150, and 250 mg/kg) have been assessed in groups encompassing three males and three females. At the dose of 250 mg/kg, all animals died within the min after the injection. At the dose of 150 mg/kg, one male died 3 min after the end of the injection. The two others showed a decreased activity and screamed when touched 15 min after the injection. Two females out of three also had these clinical symptoms at the same time point. For these five surviving animals (two males and three females), no other treatment-related clinical sign was observed till the end of the assay at day 14. At the doses of 50 and 100 mg/kg, no treatment-related sign was observed in the injected animals. These animals also showed an evolution of their weight that was similar to the one of the animals of the control group along the 14 days of the study (data not shown). Therefore, the MTD of the ABP dendrimer is 100 mg/kg in mice.

Then, we performed a second assay in another rodent species, namely Wistar rats. Three doses (50, 100, and 200 mg/kg) have been assessed in groups encompassing two males and two females. As no delayed clinical signs related to the treatment with the ABP dendrimer have been observed in the first study with mice, the study with rats stopped two days after the injection. At the dose of 200 mg/kg, one male and two females died within the min after the injection. The surviving male showed hematoma on the site of injection (tail), labored breathing, and circling. At the doses of 50 and 100 mg/kg, no treatment-related signs was observed in the injected animals. Therefore, the MTD of the ABP dendrimer is 100 mg/kg in rats.

#### 3.4.2. Repeated Injections in Rat

As the ABP dendrimer is intended for the treatment of chronic inflammatory disorders, the assessment of the MTD upon repeated injections is relevant. This study was performed in Wistar rats. Three doses (30, 60, and 120 mg/kg/day during seven consecutive days) have been assessed in groups encompassing two males and two females. At the dose of 120 mg/kg/day, one female died immediately after the fourth injection. The day before, at the third injection, this animal had slight piloerection and irregular breathing. At this dose, unsteady gait, decreased activity, and labored breathing were noted for all the animals after the first injection. The same symptomatology persisted during the entire treatment period for the surviving female. For the two males, only unsteady gait was noted after the seven and last injection. At the doses of 30 and 60 mg/kg/day, no treatment-related signs was observed in the injected animals. Body weight of these animals was not noticeably affected when compared to the animals of the control group along the eight days of the study (data not shown). Therefore, the MTD of the ABP dendrimer is 60 mg/kg/day after repeated injection for seven days in rats.

### 3.5. In Vitro Cardiac Safety

The risk of Torsade de Pointes is among the main reasons for failure in clinical phases and drug market withdrawal during the pharmacovigilance phase. Therefore, modeling cardiac electrophysiology is a key challenge to assess the safety of early preclinical drug-candidates in order to spare time and money in the drug development process [28]. Consequently, using the intracellular microelectrode technique [29], we have studied the effects of the ABP dendrimer on action potentials, and on four parameters thereof (resting potential, action potential durations at 50%, 70% and 90% of repolarization (APD_50_, APD_70_ and APD_90_, respectively), action potential amplitude, and maximal rate of depolarization dV/dt_max_). These experiments have been performed in cardiac Purkinje fibers isolated from rabbits. The effects of the ABP dendrimer have been assessed at 0.05, 0.5 and 5 µM with electric stimulation at 1 Hz and then at 0.2 Hz frequencies, on three fibers isolated from three rabbits.

**Figure 7:**
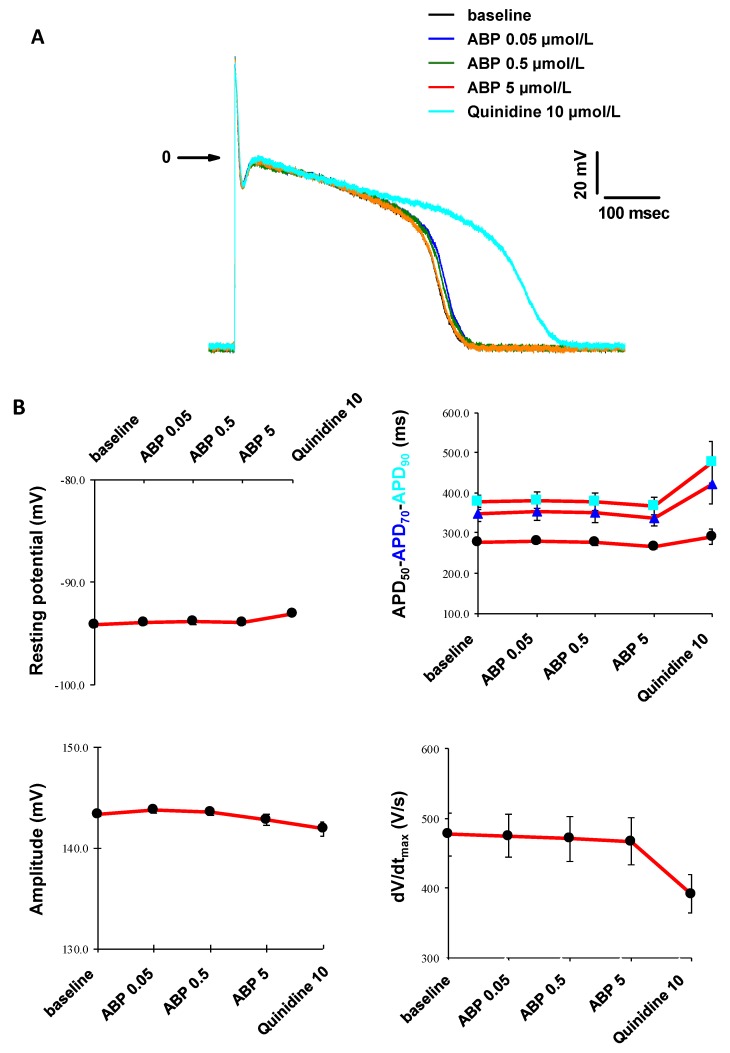
(**A**) Time-dependent effects of the ABP dendrimer at different concentrations and of Quinidine at 10 µM on the action potential at 1 Hz (one Purkinje fiber representative of three). (**B**) Effects of the ABP dendrimer at different concentrations and of Quinidine at 10 µM on the four action potential parameters at 1 Hz (Data are expressed as mean ± SEM, *n* = 3 Purkinje fibers).

**Figure 8:**
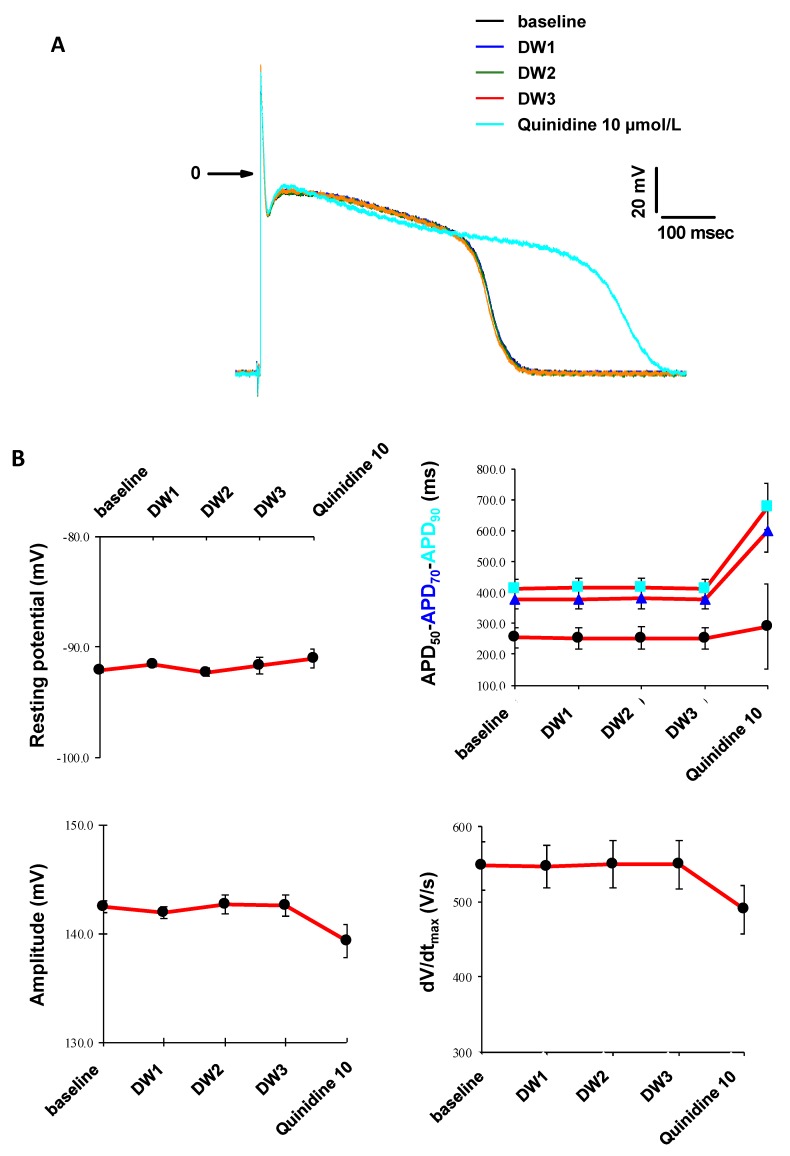
(**A**) Time-dependent effects of distilled water (DW) 0.1% and of Quinidine at 10 µM on the action potential at 1 Hz (one Purkinje fiber representative of three). (**B**) Effects of distilled water (DW) 0.1% and of Quinidine at 10 µM on the four action potential parameters at 1 Hz (Data are expressed as mean ± SEM, *n* = 3 Purkinje fibers).

During these experiments, whatever the concentrations tested and the electric stimulation, neither spontaneous automatism of the fiber preparations nor early or delayed after-depolarizations on action potentials were observed in the presence of the ABP dendrimer (Figure 7A at 1 Hz, not shown at 0.2 Hz). Distilled water (DW, the vehicle for the ABP dendrimer) was assessed as control and did not show any modification neither (Figure 8A at 1 Hz, not shown at 0.2 Hz). At both frequencies, the ABP dendrimer did not modify action potential parameters (Figure 7B at 1 Hz, not shown at 0.2 Hz), when compared to stable effects observed in the presence of distilled water (Figure 8B at 1 Hz, not shown at 0.2 Hz). For each fiber, at the end of the experiments, Quinidine (an anti-arrythmic drug) at 10 µM induced expected reverse frequency-dependent increases in APD and decreases in the maximal rate of depolarization dV/dt_max_.

## 4. Discussion

Over the years, dendrimers have been proposed for many biomedical applications such as imaging agents, nucleic acid transfection, and drug delivery. Some of them have even shown intrinsic therapeutic effects, especially against inflammatory disorders and infections [1,30]. Dendrimers are very innovating nanomolecules in the biomedical field, with promising therapeutic applications, but they are far from the standards of the pharmaceutical industry [31]. Therefore, a particular attention is paid to their potential toxicity. Few years ago we have assessed the general tolerability and toxicity, and the immuno-safety of the ABP dendrimer in non-human primates. From our point of view, this was the most relevant model to assess the immuno-safety. Nevertheless, preclinical data in non-human primates are not required by the regulatory agencies such as the American Federal and Drug Administration (FDA) or the European Medicine Agency (EMA). In order to prepare the entry of the ABP dendrimer in the regulatory preclinical phase of its development, we have gathered a series of data in rodents regarding its biodistribution after a systemic IV injection, its hematological safety, its genotoxicity, its MTD, and its early cardiac safety (Purkinje’s fibers from rabbits). To our knowledge, although many articles have reported biodistribution and safety data about dendrimers, this is the first report of a complete set of such data for a given dendrimer.

Here, we have shown that after an IV injection, the ABP dendrimer is mainly addressed to the liver and the lungs. It is also addressed in much lesser amounts to the spleen and the kidneys. For quantification purposes, the injection of a green fluorescent analogue of the ABP dendrimer has shown strong limits due to the green auto-fluorescence of tissues. Moreover, the presence of chlorophyll in the diet of the mice strengthens this green fluorescent background. Therefore, we have designed and synthesized a new analogue that emits near infrared fluorescence. This approach has enabled us to implement a quantitative follow-up of the biodistribution of the ABP-NIR dendrimer, both in isolated organs and in living animals using the IVIS Spectrum imaging system. Fluorescent labeling is a smart alternative to the use of radiolabeled compounds. Others have shown that a radiolabeled third generation (G3) PEGylated triazine dendrimer with paclitaxel groups at its surface is addressed to the liver and the spleen with a rapid decrease within two days [32], this study being a short-term one. They have also found that the triazine dendrimer is found in the kidneys rapidly after the administration and that it rapidly disappears from these organs after 24 h. We have also found only low amounts of the ABP-NIR dendrimer in the kidneys at 24 h after the injection, which precludes any renal toxicity of these molecules. Of note, the triazine dendrimer has a monomeric molecular mass of around 40 kDa and is likely aggregated in decameric structures of around 400 kDa. The ABP dendrimer (and its fluorescent analogues) has a molecular mass around 6 kDa. More recently, it was shown that a red fluorescent analogue (labeled with a Cyanine-5 dye) of a G4 PolyAmidoAmine (PAMAM) dendrimer also targets the liver in rabbits [33].

In the long-term (56 days) follow-up performed in this study, we have shown that there is no bio-accumulation of the ABP-NIR dendrimer as the total fluorescence decreases slowly with time. This slow decrease, and therefore the persistence of the ABP dendrimer in the organism, is consistent with the therapeutic efficacy at low doses demonstrated in mouse models of experimental arthritis (1 mg/kg/week for twelve weeks) and of experimental autoimmune encephalomyelitis (EAE, 10 mg/kg/week for 25 days). Although we have no pharmacokinetics data and no quantification of the half-life of the molecule, we can state that it is long enough to have efficacy at low doses in models of chronic inflammatory disorders.

Our results demonstrate the hematological safety of the ABP dendrimer in mice. The molecule does not induce hemolysis, platelet aggregation, nor disturbance in the hematological formula 24 h after administration. These data corroborate our previous results in non-human primates obtained in a longer study (four IV injections with one week intervals) [16]. However, in non-human primates we have evidenced an increase of the enzymatic activity of AST and ALT in plasma after the first injection (at the same dose). This is not found in mice, which may confirm the hypothesis that this increase observed in non-human primates was part of a transient mild inflammatory reaction due to scratching at the injection site by animals themselves. The lack of cytotoxicity of the ABP dendrimer is expected as it is ended by anionic groups. It is known that the charge and sometimes the size are crucial determinants of toxicity [34]. For instance, it was recently shown that cationic G3, G5, G7 triazine and G3, G6 PAMAM dendrimers, with a size range between 7 and 130 kDa, induce platelet aggregation in vitro at micromolar concentrations [35]. Cationic dendrimers, whatever their internal structure, are toxic due to ionic-based interactions with negatively charged biological membranes. On the contrary, neutral and negatively charged dendrimers are much more compatible for biomedical applications [36]. 

The micronucleus test has shown that the ABP dendrimer has potential genotoxicity at 215 µM. This value is 10 to 100 times higher than the active concentrations of the molecule on human monocytes in vitro (2 to 20 µM, [37]). Finally, we have shown that the MTD of the ABP dendrimer is 100 mg/kg for a single injection, and 60 mg/kg/day for repeated injections during seven consecutive days. These MTD values have to be compared with the therapeutic active doses mentioned above: 1 to 10 mg/kg/week. Taken all together, these values indicate that there is a favorable therapeutic window for the ABP dendrimer.

## 5. Conclusions

Although dendrimers have been considered for years as promising drugs and biomedical devices, their translation to clinical applications seems tedious to achieve. Among several reasons that can be more or less specific to a given family of dendrimers, one common shortcoming for the medical translation thereof is the difficulty to develop and implement robust and reliable analytical methods [38]. Validated analytical methods are mandatory to perform preclinical and clinical pharmacokinetics studies. This is one of the challenges that has to be tackled with the ABP dendrimer, together with the understanding of the molecular mechanisms of action of the molecule [39]. In conclusion, the ABP dendrimer shows a very promising safety profile. Nevertheless, most of the assays reported in this article will have to be repeated and completed in the framework of accredited Good Laboratories Practices to be compliant with the requirements of regulatory agencies before considering a first-in-man clinical phase. In addition, on the way to clinical translation, one has to remember the quotation by Howard E. Skipper, a noted American oncologist of the twentieth century: “A model is a lie that helps you see the truth”.

## Figures and Tables

**Table 1:** Results of the BN Ames’ test.

		TA 1537		TA 100		TA 98	
	Dose (µg/Dish)	Mutants/Dish	Ratio	Mutants/Dish	Ratio	Mutants/Dish	Ratio
without metabolic activation	untreated	6.5		111.5		24	
	control + ^1^	681.7	105 **	2380.3	21.3	636.3	26.5 **
	0.39	7	1.1	110.3	1	21.3	0.9
	0.78	9.7	1.5	128.7	1.2	26	1.1
	1.56	7	1.1	133	1.2	26.7	1.1
	3.13	7	1.1	132.3	1.2	32	1.3
	6.25	6.7	1	119.3	1.1	27.7	1.2
	12.5	9	1.4	126	1.1	28	1.2
	25	9.3	1.4	124.3	1.1	29.3	1.2
	50	7.7	1.2	115.7	1	33	1.4
	100	7	1.1	114.3	1	27.7	1.2
	200	11.3	1.7	123.3	1.1	37.3	1.6 **
with metabolic activation (S9 mix)	untreated	7.8		106.3		28.4	
	control + ^2^	177.3	22.7 **	1498.3	14.1 **	1686.7	59.4 **
	0.39	7.7	1	125.7	1.2	28.7	1
	0.78	7	0.9	105.3	1	35.3	1.2
	1.56	7.7	1	118.3	1.1	32.3	1.1
	3.13	7	0.9	113.3	1.1	33	1.2
	6.25	7.7	1	138.3	1.3 **	30.3	1.1
	12.5	5.3	0.7	123.3	1.2	31.3	1.1
	25	8	1	111.3	1	35.7	1.3
	50	5.3	0.7	113.7	1.1	38	1.3
	100	6.7	0.9	115.7	1.1	33	1.2
	200	4.3	0.6	127.7	1.2	26.7	0.9

^1^ without metabolic activation: For strain TA 1537, positive control is 9-aminoacridine at 1.56 µg/dish; for strain TA 100, positive control is 2-nitrofluorene at 0.5 µg/dish; for strain TA 98, positive control is sodium azide at 2 µg/dish. ^2^ with metabolic activation: For all strains, positive control is 2-anthramine at 0.5 µg/dish. ** *p* < 0.01 versus untreated.

**Table 2:** Results of the micronucleus assay on L5178Y mouse lymphoma cells.

	Dose (µM)	Relative % of Survival	Total Number of Micronuclei for 2000 Mononuclear Cells	Relative % of Survival	Total Number of Micronuclei for 2000 Mononuclear Cells
		without recovery		with recovery	
without metabolic activation	untreated	100	3	100	3
	control + ^1^	88.9	40 ***	90.8	75 ***
	430	31.1	nd ^2^	51.2	17 **
	215	79.2	9 *	99.9	12 *
	107	69.8	4	96.2	4
	53.7	80.3	3	111	nd ^2^
with metabolic activation (S9 mix)	untreated			100	3
	control + ^3^			85.1	239 ***
	773			127	4
	387			145	2
	193			119	3

^1^ without metabolic activation: Positive control is Mitomycin C at 0.05 and 0.025 µg/mL, without and with recovery, respectively. ^2^ not determined. ^3^ with metabolic activation: Positive control is Cyclophosphamide at 10 µg/mL. * *p* < 0.05, ** *p* < 0.01, *** *p* < 0.001 versus untreated.
